# Charachterization and functional analysis of scFv-based CARs to redirect T cells to IL13Rα2-positive glioma

**DOI:** 10.1186/2051-1426-3-S2-P116

**Published:** 2015-11-04

**Authors:** Giedre Krenciute, Simone Krebs, David Torres, Gianpietro Dotti, Maciej S Lesniak, Irina V Balyasnikova, Stephen Gottschalk

**Affiliations:** 1 Baylor College of Medicine, Houston, TX, USA; 2 The University of Chicago, Chicago, IL, USA; 3 Department of Pediatrics, Center for Cell and Gene Therapy, Baylor College of Medicine, Houston, TX, USA

## Background

The goal of this project is to develop T cells that express chimeric antigen receptors (CARs) as an effective immunotherapy for glioblastoma (GBM), the most aggressive, primary brain tumor in humans, which outcome remains poor. IL13Rα2 is aberrantly expressed in GBM and therefore it is a promising candidate for CAR T cell immunotherapy. While other investigators have generated IL13Rα2-targeted CARs using mutated forms of IL13 as CAR binding domains, our studies indicate that these CARs also recognize IL13Rα1, raising significant toxicity concerns. To overcome this limitation we have developed a high affinity IL13Rα2-specific scFv (M47) that does not recognize IL13Rα1 and that was used to generate scFv-based IL13Rα2-specific CAR (M47-CAR T cells) and evaluate their function.

## Methods

We constructed a panel of IL13Rα2-CARs containing M47 as an ectodomain, a short spacer or long spacer region (SSR, LSR), a CD28 transmembrane domain, and endodomains that consist of signaling domains derived from CD3ζ and co-stimulatory molecules (CD28.ζ, CD28.OX40.ζ, CD28.ζ, CD28.41BB.ζ, 41BB.ζ) IL13Rα2-CAR T cells were generated by retroviral transduction, and their effector function was compared *in vitro* and in a GBM xenograft model.

## Results

While all CARs were expressed as judged by Western blot analysis, CARs with a SSR and a CD28.41BB.ζ endodomain were not expressed on the cell surface. In cytotoxicity assays, IL13Rα2-CAR T cells only killed target cells that expressed IL13Rα2 and not IL13Rα1 confirming specificity. While all IL13Rα2-CAR T cells secreted significant levels of IFNγ in co-culture assays with the IL13Rα2+ glioma cell line U373, only CAR T cells with a short spacer region (SSR) secreted significant amounts of IL2. In contrast, T cells expressing an IL13Rα2-CAR with a deleted endodomain (M47-CAR.D) did not recognize or kill any target cells. *In vivo*, injection of IL13Rα2.SSR.CAR T cells with CD28.ζ, CD28.OX40.ζ, or 41BB.ζ endodomains into U373 -bearing mice resulted in regression of glioma xenografts and a significant survival advantage with M47-CAR.SSR.CD28.ζ T cell therapy providing the longest median overall survival (84 days vs 40 days for M47-CAR.SSR.D).

## Conclusions

T cells redirected to IL13Rα2 with M47-CARs have potent anti-tumor activity against glioma cells *in vitro*, and induce the regression of established GBM xenografts. Our study adds to the growing literature that there is an intricate interplay between scFVs, spacer region, transmembrane domain and endodomain that determine CAR function, and that there is no single optimal configuration. M47-CARs may be of value in the treatment of not only IL13Rα2-positive GBMs but also other malignancies in which IL13Rα2 is expressed.

